# Regenerative Potential of Aqueous Extract of Neem *Azadirachta indica* on the Stomach and Ileum Following Ethanol-induced Mucosa Lesion in Adult Wistar Rats

**DOI:** 10.4021/gr2010.04.173w

**Published:** 2010-03-20

**Authors:** David A. Ofusori, Benedict A. Falana, Adebimpe E. Ofusori, Ezekiel A. Caxton-Martins

**Affiliations:** a Department of Anatomy and Cell Biology, Faculty of Basic Medical Sciences, Obafemi Awolowo University, Ile-Ife, Osun-State, Nigeria; b Department of Anatomy, College of Health Sciences, Osun State University, Osogbo, Osun-State, Nigeria; c Department of Chemistry, Faculty of Science, University of Lagos, Akoka, Lagos State, Nigeria

**Keywords:** Neem *Azadirachta indica*, Stomach, Ileum, Lesion, Regeneration, Ethanol

## Abstract

**Background:**

The aim of this study was to examine whether neem *Azadirachta indica* possesses regenerative potential on the stomach and ileum at 500 mg/kg dose given every 12 hours after mucosa lesion was brought by the administration of 1 ml of 50% ethanol for 21 consecutive days in adult Wistar rats.

**Methods:**

Adult male Wistar rats used in the study were divided into 3 groups: group A received oral normal saline and served as control; group B received 1.0 ml of 50% ethanol orally every 12 hours; and group C received neem extract (500 mg/kg) orally 12 hours after ethanol (50%, 1.0 ml) administration to verify its regenerative potential. The experiment lasted for 21 days after which the animals were sacrificed following chloroform inhalation and the stomach and ileum excised and processed for histological and morphometric examinations.

**Results:**

Ethanol treated rats showed marked gross mucosal lesions in the stomach and ileum. Ulcerated mucosa with marked apoptotic bodies and destruction of glandular elements were evident in the animals (group B). Neem extract administered 12 hours after the ethanol administration showed regenerative potential against ethanol-induced mucosal damage. This was characterized by mild restoration of the ulcerated mucosa epithelium and reorganization of the cyto-architechtural outline in group C.

**Conclusions:**

Our investigation suggests that neem extract has a regenerative potential and may be adopted in the management of gastrointestinal disorders such as ulcer.

## Introduction

The neem tree *Azadirachta indica* is widely grown in Africa. Its height is about 15 meters and it bears fruits between two to three years [1, 2]. The leaves are divided into numerous leaflets, each resembling a full-grown leaf. The alternate, pinnate leaves are 20 - 40 cm (8 to 16 in.) long, with 20 to 31 medium to dark green leaflets about 3 - 8 cm long. The terminal leaflet is often missing. The petioles are short. Very young leaves are reddish to purplish in colour. The shape of mature leaflets is more or less asymmetric and their margins are dentate with the exception of the base of their basiscopal half, which is normally very strongly reduced and cuneate or wedge-shaped [1, 2].

Herbs (plant materials) as sources of medical compounds contribute to the maintenance of human health since the dawn of civilization. The role of medicinal herbs in the treatment and prevention of disease can not be overemphasized and therefore, needed to be properly investigated to guide its usage and application. Herbal medicine manufacturers all over the world are undergoing intensive research aimed at producing drugs that cure stomach lesions such as ulcers. Neem has been adopted for the management of stomach and intestinal problems [1]. Anti-inflammatory properties of neem provide relief to stomach lesions. It is now considered as a valuable source of unique natural products for development of medicines against various diseases and also for the development of industrial products [3, 4]. Whether drinking a simple cup of tea out of the leaves or taking extracts of the neem leaf, neem significantly and consistently reduced insulin requirements for nonkeytonic, insulin fast, and insulin sensitive forms of Diabetes. The pain, inflammation, and swelling of the joints in arthritis can be greatly reduced by different compounds in neem. A significant antiulcer effect was observed with nimbidin (an important phytochemical in neem) in preventing acetylsalicylic acid, indomethacin, stress or serotonin-induced gastric lesions as well as histamine or cysteamine-induced duodenal ulcers [5, 6]. Azadirachta indica extract (100 - 800 mg/kg p.o., 100 - 25 mg/kg i.p.) significantly inhibited gastric ulceration induced by indomethacin [7]. Neem leaf extract was also reported to prevent *OH-mediated mucosal DNA damage in vitro by scavenging the *OH, thus, offering antiulcer activity by blocking acid secretion through inhibition of H+-K+-ATPase and by preventing oxidative damage and apoptosis [8].

Very little work has been done on the medicinal applications of *Azadirachta indica* and hence extensive investigation is needed to exploit their therapeutic potentials in the management of diseases. Some works have been done on the protective effect of neem extract [9, 10]. Its regenerative potential is yet to be completely elucidated most especially in the gastrointestinal tract [9, 10].

There is therefore need to examine the regenerative potential of aqueous neem extract *Azadirachta indica* on the mucosa of the stomach and ileum in adult Wistar rats.

## Materials and Methods

### Preparation of extract

Leaves from neem tree were procured and authenticated by a taxonomist in the Botany Department of the Obafemi Awolowo University, Nigeria. A voucher was deposited at the herbarium. The leaves were air dried and about 100 g of powder was extracted with distilled water. The aqueous extract was completely dried under vacuum and then weighed and the residue was used in testing (a yield of 39%). The dried extract was dissolved in normal saline before gavages.

### Animals

Thirty adult male Wistar rats weighing 185 - 200 g were fed with standard pellet diet (Ladokun feeds Ltd, Ibadan, Nigeria) and were provided water *ad libitum*. Animals were housed under standard environmental conditions. All animals were treated in accordance with the ‘Guide for the Care and Use of Laboratory Animals’ prepared by the National Academy of Sciences and published by the National Institutes of Health [11].

### Experiment design

The control group (group A; n = 10) received oral normal saline. One ml of 50% ethanol was used orally to produce mucosa lesion in group B (n = 10). In another group (group C; n = 10), the rats were administered with 500 mg/kg of neem extract 12 hours after ethanol treatment. All the animals were fed with pelleted diet and water *ad libitum* throughout the experimental period (21 consecutive days). Twenty-four hours after the last administration, the animals were sacrificed under chloroform anaesthesia. Subsequently, the stomach and ileum were excised. The stomachs were opened along their greater curvature and gently rinsed under running tap water and spread on a paraffin plate. Lesions in the glandular part of the stomach were observed macroscopically.

### Histological procedure

Samples from the stomach and ileum of all the sacrificed animals were fixed in 10% formol saline and processed with paraffin for histological examination. Serials section at 5 µm thickness was obtained on a rotary microtome and stained with Haematoxylin and Eosin (H and E).

### Morphometric analysis

The stained sections were subjected to morphometric analysis recommended by World Health Organization [12] which included: dividing the eye piece occulometer into two 100 small divisions, the stage micrometer scale was made up to 1 mm divided into 0.1 mm divisions and each 0.1 mm was divided into 0.01 mm, the eye piece scale (occulometer) was inserted into the eye piece of the microscope by removing the superior lens thus placing the scale on the field stop, the stage micrometer was also placed on the stage of the microscope, the stage scale was focused by the low power objective lens (x 4), the stage and the eye piece scales were adjusted until there was a parallel point between the two scales, the number of the eye piece divisions and its corresponding stage measurements was noted. The occulometer fixed into the Olympus Microscope was then focused through stained sections of the stomach and ileum to allow for the measurement of the thicknesses of the mucosa and muscularis externa.

### Statistical analysis

Data were expressed as Mean ± Standard Error of Mean (S.E.M). For comparison between two means, student’s t-test was used, and for multiple comparisons, ANOVA was used, to determine the level of significance. Except where otherwise stated, p < 0.05 was taking as the significant level.

## Results

**Figure 1:**
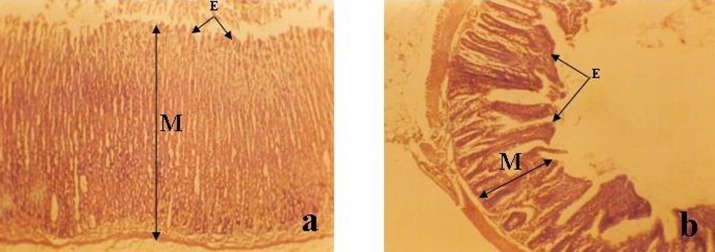
Photomicrograph of (a) Stomach (b) Ileum of ethanolic-treated group (group B) (Mag. x 100). Note the disorganization of the mucosa (M) and the ulceration of the epithelium (E) in (a) and (b) as compared with Fig. 2.

**Figure 2:**
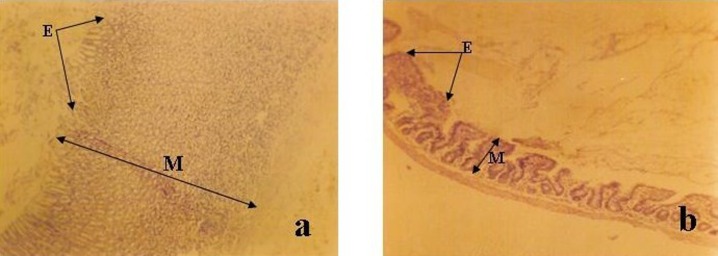
Photomicrograph of (a) Stomach (b) ileum of control (group A) (Mag. x 100). Note the well organized mucosa (M) and well preserved epithelium (E) in (a) and (b).

**Figure 3:**
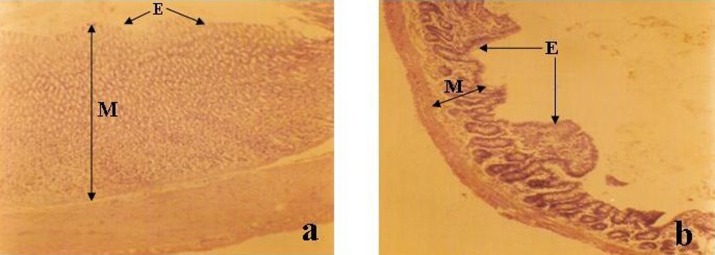
Photomicrograph of (a) Stomach (b) Ileum of treated (group C) (Mag. x 100). Note the distinct glandular elements of the mucosa (M) and the restoration of the mucus and epithelium (E) in (a) and (b) as compared with Fig. 2.

The result showed that ethanol treated rats was marked by gross mucosal lesions in the stomach and ileum. Ulcerated mucosa with marked apoptotic bodies and destruction of glandular elements coupled with a significant reduction in the mucosa and muscularis externa (p < 0.05) were evident in the animals (group B) vis-a-vis control animals (Fig. 1, 2). Neem extract administered 12 hours after the ethanol administration for 21 consecutive days showed regenerative potential against ethanol-induced mucosal damage (Fig. 3). This was characterized by mild restoration of the ulcerated mucosa epithelium and reorganization of the cyto-architechtural outline in the animals (group C). Also, there was a significant increase in the thicknesses of the mucosa and muscularis externa (p < 0.05) when group C was compared with group B (Tables 1, 2).

**Table 1:** Showing the Micromorphometry of the Stomach (M ± SEM)

Measured Parameters	Control(Group A)	1.0 ml of 50% Ethanol (Group B)	1.0 ml of 50% Ethanol + 500 mg/kg Neem (Group C)
Stomach Mucosa (µm)	776 ± 25.37	526 ± 23.01*	602 ± 22.20*§
Stomach Muscularis externa (µm)	528 ± 24.24	311 ± 21.11*	401 ± 22.22*§

n = 5; *P < 0.05 (significant) vs. control; §P < 0.05 (significant) group B vs. group C.

**Table 2:** Showing the Micromorphometry of the Ileum (M ± SEM)

Measured Parameters	Control(Group A)	1.0ml of 50% Ethanol (Group B)	1.0ml of 50% Ethanol + 500 mg/kg Neem (Group C)
Ileum Mucosa (µm)	281 ± 31.43	101 ± 29.42*	212 ± 29.58*§
Ileum Muscularis externa (µm)	63 ± 11.16	41 ± 8.11*	54 ± 10.13*§

n = 5; *P < 0.05 (significant) vs. control; §P < 0.05 (significant) group B vs. group C.

## Discussion

Our present investigation has demonstrated that aqueous extract of neem *Azadirachta indica* possesses regenerative potential at the 500 mg/kg dose given 12 hours after the administration of 1 ml of 50% ethanol for 21 consecutive days. The significant increment observed when the thicknesses of the mucosa and muscularis externa of the stomach and ileum in group C were compared with animals in group B (p < 0.05; Tables 1, 2), clearly demonstrates the regenerative capacity of *Azadirachta indica.* Modern clinical studies have identified a number of compounds in the neem tree that effectively regulate immune system functions. Flavonoid in Azadirachta indica is highly rich in antioxidants [8]. The mechanism of action is believed to be triggered by the presence of these antioxidants. Antioxidants have been known to mop up free radicals in living tissues [13, 14]. The antioxidant properties of *Azadirachta indica* may have assisted in scavenging the free radicals generated by the presence of ethanol thus, leading to the gradual restoration of the muscular coats. This antioxidant effect may also have a role to play in the mild restoration of the ulcerated mucosa epithelium and reorganization of the cyto-architechtural outline in the animals administered with 500 mg/kg dose of the extract 12 hours after the administration of 1 ml of 50% ethanol for 21 consecutive days (Fig. 1-3). Administration of 30 to 60 mg of freeze-dried neem bark extract twice per day led to a significant reduction in stomach acid levels and near complete healing of all people with duodenal ulcers over 10 weeks time in a preliminary clinical trial [15, 16]. Bandyopadhyay et al [15] also reported that the extract administered for 10 days at the dose of 30 mg caused a significant decrease in gastric acid secretion. The volume of gastric secretion and its pepsin activity were noted to be inhibited by 63% and 50%, respectively.

The actual mechanism for our observation is yet to be understood but may be connected with replacement of mast cell granules [16], reduction in gastric acid secretion and gradual deposition of mucus on the gastrointestinal walls, thus protecting the epithelial layer of the stomach from gastric acid digestion as observed in Fig. 3. Earlier studies have shown that restoration of damaged gastrointestinal epithelium improves the formation of gastrointestinal mucus which protects the mucosa against necrotizing agents [17, 18]. In our present investigation, the regeneration of epithelial lining and mucus deposition in the stomach and ileum were observed to occur concomitantly. Previous animal studies had shown that neem bark works by protecting the stomach's mucous lining and preventing oxidative damage by blocking lipid peroxidation and scavenging the free radicals that are a major cause of ulcers [19]. Garg et al [16] concluded that neem dose-dependently reduced gastric ulcer severity in rats subjected to stress and also decreased ethanol provoked gastric mucosal damage.

More research is needed to corroborate this investigation most especially the pharmacological implications.

Our investigation suggests that neem extract has a regenerative potential and may be adopted in the management of gastrointestinal disorders such as ulcer.
